# Quantifying the quality of hand movement in stroke patients through three-dimensional curvature

**DOI:** 10.1186/1743-0003-8-62

**Published:** 2011-10-31

**Authors:** Rieko Osu, Kazuko Ota, Toshiyuki Fujiwara, Yohei Otaka, Mitsuo Kawato, Meigen Liu

**Affiliations:** 1 Computational Neuroscience Laboratories, Advanced Telecommunications Research Institute International (ATR), Kyoto, Japan; 2 Department of Rehabilitation Medicine, Keio University School of Medicine, Tokyo, Japan; 3 Department of Rehabilitation Medicine, Tokyo Bay Rehabilitation Hospital, Narashino, Japan

## Abstract

**Background:**

To more accurately evaluate rehabilitation outcomes in stroke patients, movement irregularities should be quantified. Previous work in stroke patients has revealed a reduction in the trajectory smoothness and segmentation of continuous movements. Clinically, the Stroke Impairment Assessment Set (SIAS) evaluates the clumsiness of arm movements using an ordinal scale based on the examiner's observations. In this study, we focused on three-dimensional curvature of hand trajectory to quantify movement, and aimed to establish a novel measurement that is independent of movement duration. We compared the proposed measurement with the SIAS score and the jerk measure representing temporal smoothness.

**Methods:**

Sixteen stroke patients with SIAS upper limb proximal motor function (Knee-Mouth test) scores ranging from 2 (incomplete performance) to 4 (mild clumsiness) were recruited. Nine healthy participant with a SIAS score of 5 (normal) also participated. Participants were asked to grasp a plastic glass and repetitively move it from the lap to the mouth and back at a conformable speed for 30 s, during which the hand movement was measured using OPTOTRAK. The position data was numerically differentiated and the three-dimensional curvature was computed. To compare against a previously proposed measure, the mean squared jerk normalized by its minimum value was computed. Age-matched healthy participants were instructed to move the glass at three different movement speeds.

**Results:**

There was an inverse relationship between the curvature of the movement trajectory and the patient's SIAS score. The median of the -log of curvature (MedianLC) correlated well with the SIAS score, upper extremity subsection of Fugl-Meyer Assessment, and the jerk measure in the paretic arm. When the healthy participants moved slowly, the increase in the jerk measure was comparable to the paretic movements with a SIAS score of 2 to 4, while the MedianLC was distinguishable from paretic movements.

**Conclusions:**

Measurement based on curvature was able to quantify movement irregularities and matched well with the examiner's observations. The results suggest that the quality of paretic movements is well characterized using spatial smoothness represented by curvature. The smaller computational costs associated with this measurement suggest that this method has potential clinical utility.

## Background

Stable manipulation of objects, for instance in activities such as raising a glass of water to the mouth, requires smooth control of the hand. Hemiparesis of the arm following stroke results in a degradation in the quality of hand movements. To measure the level of impairment in stroke patients with hemiparesis a number of assessment tools are available, including the Brunnstrom stage for motor impairment [1], the Motricity Index [2], the Fugl-Meyer assessment [3] and the Stroke Impairment Assessment Set (SIAS) [4-6]. Of the scaled assessments available, the psychometric properties of the SIAS (which was developed in and is frequently used in Japan) are well described, with this scale providing the ability to evaluate arm function based on the observed clumsiness of movement [4-6].

To motivate stroke patients to use their paretic arm [7-10], it is important that the affected arm can execute a task quickly and smoothly. Therefore, movement free of clumsiness is an important characteristic of movement kinematics, and may promote the use of the paretic arm [11]. Movement irregularity represented by clumsiness may include both spatial and temporal aspect of trajectory smoothness. Quantitative evaluation of clumsiness, or spatio-temporal irregularity, is considered helpful. However, existing scales, including the SIAS scale, are based on the examiner's observations and thus may be subject to subjectivity or observer bias. This has prompted research into the development of a process that allows for the objective evaluation of movement based on the analysis of movement kinematics [12-16]. It is also important to determine if the clinical scale of movement irregularity obtained through observation correlates with the objective measures of movement irregularity [17-23].

Research into the field of computational motor control has shown that well-trained movements are smoothest in either the kinematic domain or the motor command domain [24-26]. Based on these observations, attempts have been made to evaluate movement based on smoothness, normally expressed as the presence of jerkiness (rate of change of acceleration), in the healthy participants. For example, Hogan and Sternad proposed a mean squared jerk measure normalized by the minimum possible mean squared jerk of that movement amplitude and duration [27], which is called the mean squared jerk ratio (MSJ ratio). The MSJ ratio is one of the dimensionless jerk-measures occurring independent of movement duration and amplitude [28]. In patients with conditions such as stroke, movement is typically characterized by many sub-movements [29-31]; therefore, it is expected that in these patients movement will be jerkier than in healthy people. Motor control researchers have attempted to incorporate some form of jerk measure into the functional evaluation of patients with stroke-induced deficits or other motor deficits [32-35].

In this study, in addition to jerk metrics, we focused on three-dimensional curvature, mathematically described as an inverse of the radius of curvature at the each point on the trajectory, to evaluate the quality of hand movement. Curvature and jerk differ in the sense that curvature quantifies spatial characteristics, while jerk quantifies the temporal characteristics of trajectory. In theory, curvature is always zero for movement on a straight path even when the amount of jerking is high. Therefore, in theory, the curvature metric and the jerk metric do not correlate with each other. However, in reality, the human movement path is not perfectly straight except when the movement path is constrained by a physical object. When an abrupt change in acceleration (stop or reversal of the movement) occurs, the path will also sharply curve, resulting in high curvature [36,37]. Jerk requires a third order derivative of position, while curvature can be computed using first-order (velocity) and second-order (acceleration) derivatives.

In healthy participants, a reaching movement is ballistic and curvature is generally small in the middle, at around 0.01 (1/mm) or less [37]. Curvature increases only around the posture phase of a discrete movement or the reflecting point of rhythmic movement. Here, we hypothesized that, in stroke patients, the curvature increases even in the middle of reaching due to the patient's inability to control the movement and the existence of sub-movements. In this study, we tested whether the irregularity of movement can be quantified by curvature metrics, by evaluating movement in the paretic arm of stroke patients, against the movement of age-matched healthy volunteers. We then compared our recorded metrics with the SIAS score and upper extremity subscales of the Fugl Meyer Assessment, as well as with previously proposed jerk metrics. Finally, we examined how the curvature and jerk metrics are sensitive to the movement speed.

## Methods

### Participants

Sixteen patients suffering from hemiparesis were recruited into the study. The thirteen patients participated in Experiment 1 were drawn from a larger group who were hospitalized in a university hospital for 3 weeks for the purpose of intensive training to improve finger extension movement through the HANDS therapy [9]. These patients (P1-P13) were expected to obtain major improvements in hand function (as evaluated using the SIAS finger function test score). However, the HANDS therapy was not targeting proximal upper extremity function, which is the process involved in reaching movements and what we were assessing in this study (see below). As the aim of this study was to evaluate the movement kinematics of these patients, and not to evaluate the HANDS therapy, we did not feel that the inclusion of patients from the HANDS trial would affect, or bias, our findings. To be recruited into this study, patients had to meet the following inclusion criteria: (1) the time since stroke onset was longer than 150 days; (2) the patient had no cognitive deficits; (3) there was no pain in the paretic upper extremity; (4) the passive extension range of motion was greater than 0 degrees in the affected wrist and -10 degrees at the metacarpophalangeal (MP) joints. In the patients recruited into the study it was confirmed through outpatient consultation before admission that there were no detectable motor improvements in the last month. The three additional patients (P14, P15, P16) who participated in Experiment 2 were outpatients recruited through the Tokyo Bay Rehabilitation Hospital. These three patients also met the above inclusion criteria. Nine right-handed healthy volunteers free of orthopedic or neurological disorders were also recruited into the study. One of these volunteers participated in the Experiment 1 (H1, a 38-year-old female), The other eight (H2-H8, aged from 23 to 62, four male and four female) participated in Experiment 2. The purpose of the study was explained to all of the participants and informed consent was obtained from all participants. The study was approved by the institutional ethics committee.

### Tasks

In Experiment 1, the patients were asked to grasp a plastic glass with the hand of the affected side. The patients were then asked to move the glass from the lap to the mouth and back to the lap repeatedly for 30 s at a comfortable speed using the shoulder, elbow and wrist joints. The position of the glass was measured with a sampling rate of 200 Hz using an OPTOTRAK Certus (see APPENDIX). The measurements were performed twice. The initial measurement was just after admission and the final measurement was just before discharge. The period between the initial and final measurements was approximately 3 weeks. The healthy participant's left arm movement (H1) was also measured twice in the same manner as the stroke patients. In Experiment 2, the participants were asked to execute movements in the three different patterns. In the first pattern, the movements were executed continuously at a comfortable speed as in Experiment 1 (comfortable condition). In the second pattern, the movements were executed continuously at maximum speed (fast condition). In the third pattern, the movements were executed slowly (slow condition). The eight healthy participants were asked to move either their left or right arm. The three patients were first asked to move the unaffected arm and then asked to move the affected arm. Thus in the analysis, we treated the unaffected side movement of the three patients as healthy arm data. Consequently, we acquired data from 11 unaffected arms (mean 53.5 years; SD 14.1 years) age matched with the paretic arms participated in Experiment 1 and the left arm (from 5 participants) and right arm (from 6 participants) were counterbalanced among participants. Three of the healthy participants (H2, H3, H4) worked in the rehabilitation profession (as an occupational therapist, physiotherapist and rehabilitation doctor) and these participants were also asked to mimic the movement of stroke patients (mimic condition). The position measurement was carried out in the same way as in Experiment 1.

### Clinical assessments

For Experiment 1, the patients movement was assessed using a number of tests: the SIAS upper extremity motor function assessment, the upper extremity subsection of the Fugl-Meyer Assessment, and the modified Ashworth scale (MAS) at elbow joint. These tests were performed at the time of admission and discharge by two board-certified physiatrists, who were independent of and blinded to the study. The SIAS motor function assessment has been shown to strongly correlate with both the Motricity Index and Brunnstrom stage [6]. The SIAS upper extremity motor function assessment has two components: 1) the Knee-Mouth test, which evaluates proximal function, and 2) the Finger test that evaluates individual finger movements. In this study, we focused on the Knee-Mouth test because reaching movements mainly involve the proximal joints (see APPENDIX). The Knee-Mouth test is rated from 0 to 5, with 0 indicating complete paralysis and 5 indicating no paralysis. The scores 3, 4, and 5 are rated according to the observed smoothness in the movement trajectory (severe or moderate clumsiness rating a score of 3, mild clumsiness rating 4, and smoothness comparable to the unaffected side rating 5). The differences among scores 1, 2 and 3 reside in the patient's ability to raise their arm to a particular height (up to mouth for 3, up to nipple for 2, lower than the nipple for 1), irrespective of the smoothness of the movement trajectory. Within the upper extremity subscale of Fugl-Meyer Assessment, the total score of the following sub-items were used in this study (FMA-UE); flexor synergy, extensor synergy, movement combining synergies, movement out of synergy, wrist, and hand. The total possible score for this test was 54.

### Analysis

The acquired position data was digitally low pass filtered (with a Butterworth filter) with a cut off frequency of 8 Hz since a movement fluctuation higher than 8 Hz may be caused by other factors such as tremor. For the analysis, we used the portion of the position data where the movement pattern was relatively stable and did not include measurement error (missing data caused by occlusion of the marker from the camera because of the unexpected pronation of several patients), which was 15 s for Experiment 1 and 25 s for Experiment 2. The position data was then rotated so that the main movement direction (from table to mouth) corresponded to the x-axis. Velocity and acceleration was computed by two point numerical differentiation.

#### Curvature and MedianLC (median of -log of curvature)

The three-dimensional instantaneous curvature at each time point was computed based on the following equation.

(1) $$\kappa^{2}=\frac{1}{\rho^{2}}=\frac{\left({\dot{x}}^{2}+{\dot{y}}^{2}+{\dot{z}}^{2}\right)\left({\ddot{x}}^{2}+{\ddot{y}}^{2}+{\ddot{z}}^{2}\right)-{\left(\dot{x}\ddot{x}+\dot{y}\ddot{y}+\dot{z}\ddot{z}\right)}^{2}}{{\left({\dot{x}}^{2}+{\dot{y}}^{2}+{\dot{z}}^{2}\right)}^{3}}$$

Because the distribution of instantaneous curvature is skewed, we computed the -log of the curvature (-log(κ)). Next, the -log(κ) at the time point when the movement speed (tangential velocity) exceeds 50 mm/s was extracted. The median -log(κ) at all extracted time points was computed as a representative of that trajectory, and designated MedianLC.

#### Jerk and MedianLJ (median of log of jerk)

Jerk at each time point was computed according to the following equation,

(2) $$J={\left({\overset{\mathrm{...}}{x}}^{2}+{\overset{\mathrm{...}}{y}}^{2}+{\overset{\mathrm{...}}{z}}^{2}\right)}^{\frac{1}{2}}$$

Because the distribution of jerk is skewed, we took the log of the jerk (log(*J*)). The median of log(*J*) was computed as a representative of that trajectory, which was designated MedianLJ. The portion of movement was extracted using the same threshold of 50 mm/s in movement speed (tangential velocity) as in MedianLC when computing median of the distribution.

#### Mean squared jerk ratio (MSJ ratio)

We computed the MSJ ratio, which is the mean squared jerk normalized by its minimum value [27].

(3) $$\begin{array}{c} \text{MSJ}\;\text{ratio}=\frac{MeanJ^{2}}{Mean{J^{2}}_{0}}\\ MeanJ^{2}=\frac{1}{d}\overset{t_{f}}{\underset{t_{0}}{\int }}J^{2}\\ Mean{J^{2}}_{0}=\frac{360A^{2}}{d^{6}} \end{array}$$

where *A *denotes movement amplitude and *d *denotes movement duration.

Assuming that discrete movements were concatenated, each discrete movement segment that includes a single stroke was identified from continuous movement data, with a threshold of 10% of the maximum speed of those data. The movement duration and amplitude of each segment was computed for normalization. The log of the MSJ ratio was averaged across segments for each participant. The portions where segmentation was not successful (such as a segment with an amplitude smaller than 0.1 m) were excluded from analysis. The average number of extracted movement segments across participants was 8.15 ± 2.97. Since we could not successfully segment the movement of patient 4 because his movements were continuous, we excluded this patient's data from this analysis.

### Statistics

For correlation analysis, Spearman's ranked correlation coefficient was applied. For the comparison among groups, a Kruskal-Wallis test was applied. Consistency and reliability of the measure was assessed by intraclass correlation coefficient (ICC).

## Results

### Clinical characteristics of the patients involved in the study

Patient clinical characteristics are described in Table 1. The average age of the patients in Experiment 1 was 53.7 ± 15.0 years (range: 26 - 72 years). The median SIAS Knee-Mouth test score at admission was 3, with a range from 2 to 4 (Table 2). Patients with a score of 0 or 1 were not included. Although the HANDS therapy targeted improvement of finger function, patients 3, 4 and 5 showed an improvement in the SIAS Knee-Mouth test score, whereby their score improved from 2 to 3 during hospitalization [9]. This means that these patients were not able to touch the mouth at admission, but were able to at discharge. The median of SIAS Knee-Mouth test score at discharge was 3.

**Table 1:** Patient Clinical Characteristics

Patient ID	Age (years)	Sex	Affected side	Days from onset	Lesion type	Lesion location
Experiment 1						
P1	65	F	R	780	CI	corona radiata
P2	42	M	R	4170	CI	corona radiata
P3	72	M	R	1800	CI	MCA
P4	60	M	L	1140	CI	MCA
P5	60	M	L	990	CI	basal ganglia
P6	67	F	R	2675	CH	basal ganglia
P7	70	M	R	210	CI	medulla oblongata
P8	52	M	L	2160	CI	N/A
P9	26	M	L	420	CH	sub-cortical hematoma
P10	49	M	R	360	CI	N/A
P11	58	F	R	612	CH	thalamus
P12	26	M	L	2700	CI	MCA
P13	51	M	R	315	CH	basal ganglia

AVG/count	53.7	10M/3F	8R/5L	1410	9CI/4CH	

(SD)	(15.0)			(1211)		
Experiment 2						
P14	67	F	L	1110	CH	thalamus
P15	58	M	R	1418	CH	thalamus
P16	72	M	L	624	CI	corona radiate

F, female; M, male; R, right; L, left; CI, cerebral infarction; CH, cerebral hemorrhage; AVG, average; SD, standard deviation; MCA, middle cerebral artery; N/A, not available.

**Table 2:** Comparison between the MedianLC and log of MSJ ratio with other functional assessment scores

	Initial measurement					Final measurement				
**Patient ID**	**SIAS K-M**	**FMA-UE**	**MAS elbow**	**MLC**	**LMSJR**	**SIAS K-M**	**FMA-UE**	**MAS elbow**	**MLC**	**LMSJR**

P1	2	15	1	4.36	11.13	2	19	0	4.41	10.08
P2	2	21	1+	3.90	10.48	2	27	1	4.29	9.57
P3	2	22	1+	3.68	7.52	3	30	1	4.13	8.49
P4	2	33	1	4.26	N/A	3	37	1	4.61	N/A
P5	2	30	1	3.99	10.47	3	39	1	3.71	10.69
P6	3	17	2	4.33	9.58	3	28	1	4.49	8.27
P7	3	32	1+	5.11	7.66	3	45	1	4.66	9.46
P8	3	36	3	5.12	8.29	3	43	1+	4.91	7.58
P9	3	31	1	4.50	8.48	3	35	0	5.06	7.48
P10	4	N/A	N/A	5.10	7.20	4	N/A	N/A	4.93	7.93
P11	4	50	2	4.73	8.03	4	50	1+	4.63	8.52
P12	4	48	1	5.57	6.02	4	52	0	5.54	5.43
P13	4	51	1	5.11	5.73	4	53	0	5.21	5.86
H1	(5)	(54)	(0)	5.74	6.37	(5)	(54)	(0)	5.80	5.69

SIAS K-M, Stroke Impairment Assessment Set Knee-Mouth test; FMAUE, Fugl Meyer Assessment of the upper extremity (where a total score of 54 points was possible); MAS, modified Ashworth scale; MLC, medial of log of curvature (MedianLC); LMSJR, log of mean squared jerk ratio.

### Characteristics of hand path movement

**Figure 1:**
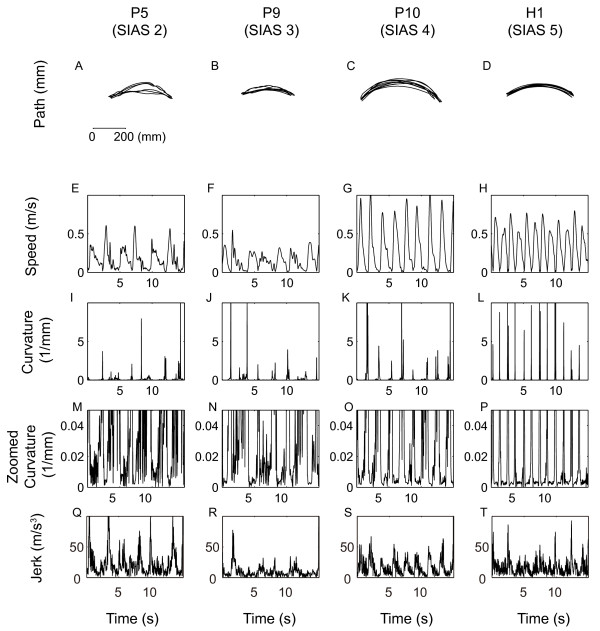
**Hand paths, including the speed, curvature, and jerk profiles were evaluated in four representative participants**. Panels A, B, C and D show the respective hand paths. The hand path is projected on a plane composed of the first principal component (main movement direction: left to right correspond to table to mouth) and the second principal component (lower side in general corresponds to being proximal while upper corresponds to being distal from the body). Panels E, F, G, and H show speed (tangential velocity); panels I, J, K, and L show curvature profiles for the patients with SIAS scores of 2 (patient P5), 3 (patient P9), 4 (patient P10), and the healthy volunteer (H1), respectively. Panels M, N, O, and P show the same curvature profiles as in panels I, J, K, and L, but are zoomed around the low curvature values between 0 and 0.05 (1/mm). Panels Q, R, S, T show the jerk profiles computed by Equation (2).

Figure 1 shows the initial measurements for hand path, speed, curvature and jerk movement in the patients with a SIAS score of 2, 3, and 4, and in the healthy participant H1 respectively. The hand path and speed profiles demonstrated decreased irregularity as the SIAS score increased. When focusing on the curvature around its smaller value (zoomed curvature), the difference was conspicuous since the curvature dropped to a very small value and remained less than 0.005 (1/mm) in the healthy volunteer (H1), but tended to fluctuate in the stroke patients. Especially for those patients who had lower SIAS scores (e.g., patients who scored 2 or 3), the curvature remained high even in the middle of the movement. However, jerk was not consistent across the SIAS scores. This is probably because jerk increases not only with movement irregularity but also with movement speed, suggesting the necessity of normalization.

### Distribution of the -log(κ) and log(J)

**Figure 2:**
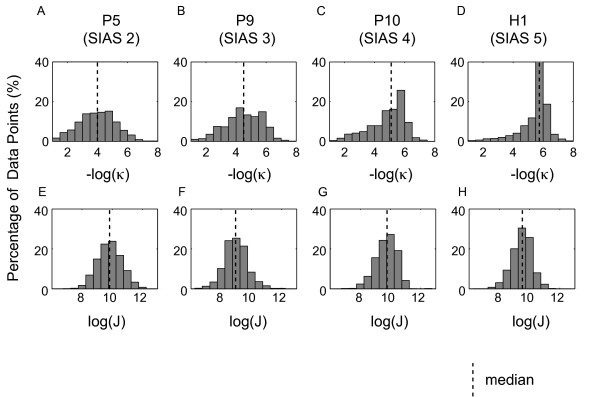
**Histograms demonstrating the -log(κ) and log(*J*)**. Panels A, B, C, and D show the -log(κ) expressed as a percentage of data points in the extracted movement strokes for patients with SIAS scores of 2 (patient P5), 3 (patient P9), 4 (patient P10), and a healthy volunteer (H1), respectively. The vertical dashed lines denote the median of the distribution. Panels E, F, G and H show the log(*J*) as described above.

The upper panels of Figure 2 show the -log(κ) during the movement for the participants with a SIAS score of 2, 3, and 4 and the healthy participant H1 (those described in Figure 1). As the SIAS score increased, the median of the -log(κ) (MedianLC; vertical dashed line) shifted to the right, suggesting that the number of the data points with a lower curvature increased. In Experiment 1, the MedianLC in the initial measurements was significantly different in the three SIAS score groups (Kruskal-Wallis test, *p *< 0.05), and post-hoc testing revealed that the MedianLC of the SIAS 3 and 4 groups was significantly higher than the MedianLC of the SIAS 2 group (Wilcoxon test, *p *< 0.05). The median of MedianLC for the respective SIAS score groups was as follows: SIAS 2 group, 3.99 (five patients); SIAS 3 group, 4.81 (four patients); SIAS 4 group, 5.11 (four patients) (Table 2). The MedianLC in the initial measurement for the healthy participant, H1, was 5.74. However, as shown in the lower panels of Figure 2, there was no significant relationship between the MedianLJ and the SIAS score. The Spearman ranked correlation coefficient between the initial MedianLJ and the initial SIAS score was -0.099 (*p *= 0.736) and that between the final MedianLJ and the final SIAS score was -0.145 (*p *= 0.621).

### Correlation between the MedianLC, MSJ ratio and clinical assessment scores

**Figure 3:**
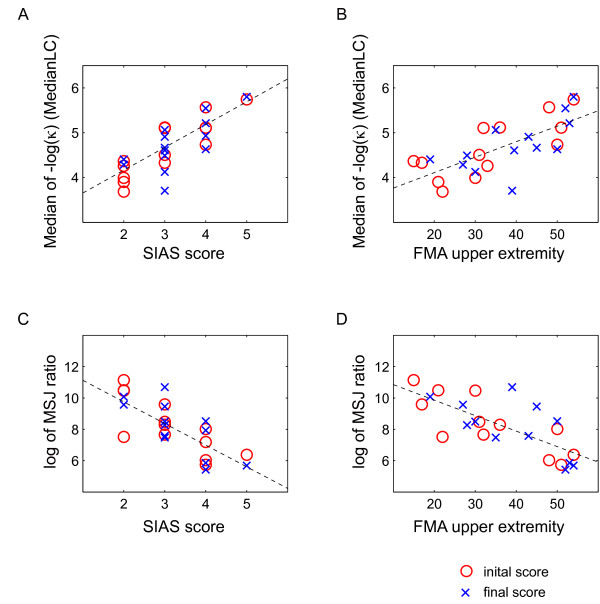
**The relationship between the MedianLC or the MSJ ratio and the different clinical assessment scores**. Magenta circles denote initial measurements while blue crosses denote the final measurements for the 13 patients and the healthy volunteer, H1, who participated in Experiment 1. Panel A plots the MedianLC against the SIAS scores. Panel B plots the MedianLC against the FMA-UE (where a total score of 54 points was possible). Panel C plots the log of MSJ ratio against the SIAS scores. Panel D plots the log of the MSJ ratio against FMA-UE. The dashed line shows the linear fitting of the data represented by the magenta circles and blue crosses.

We analyzed the correlation between the MedianLC and clinical assessment scores in Experiment 1. Figure 3A plots the MedianLC against the SIAS score and these two variables were correlated. The Spearman ranked correlation coefficient for the initial MedianLC and SIAS was 0.842 (*p *< 0.001; magenta circles), whereas the correlation between the final MedianLC and SIAS was 0.733 (*p *< 0.005; blue crosses). Figure 3B plots the MedianLC against the FMA-UE score and these two variables were correlated. The Spearman ranked correlation coefficient for the initial MedianLC and FMA-UE was 0.753 (*p *< 0.005; magenta circles), whereas the correlation between the final MedianLC and FMA-UE was 0.747 (*p *< 0.005; blue crosses).

Since the MedianLJ was not correlated with the SIAS score, we computed the MSJ ratio, which represents the jerk normalized with the minimum possible jerk of the corresponding movement amplitude and duration (Table 2). Figure 3C plots the log of MSJ ratio against the SIAS scores. The Spearman ranked correlation coefficient between the initial log of the MSJ ratio and the SIAS was -0.769 (*p *< 0.005; magenta circles), while the correlation between the final measurements was -0.7 (*p *< 0.01; blue crosses). Figure 3D plots the log of the MSJ ratio against the FMA-UE scores. The Spearman ranked correlation coefficient between the initial log of the MSJ ratio and the FMA-UE was -0.797 (*p *< 0.005; magenta circles), while the correlation between the final measurements was -0.643 (*p *< 0.05; blue crosses).

Neither the MedianLC nor the log of the MSJ ratio significantly correlated with the MAS elbow scores, suggesting that these variables do not represent the spasticity at elbow joint. We then compared the MedianLC with the log of the MSJ ratio. The Spearman ranked correlation coefficient between the MedianLC and the log of the MSJ ratio was -0.659(*p *< 0.05) for the initial measurements and -0.895 (*p *< 0.0001) for the final measurements. The significant correlation between these variables demonstrates that in stroke patients the spatial smoothness, represented by MedianLC, is related to temporal smoothness, represented by jerk.

### Experiment 2: Distribution of the -log(κ) and MSJ ratio for different movement patterns

**Figure 4:**
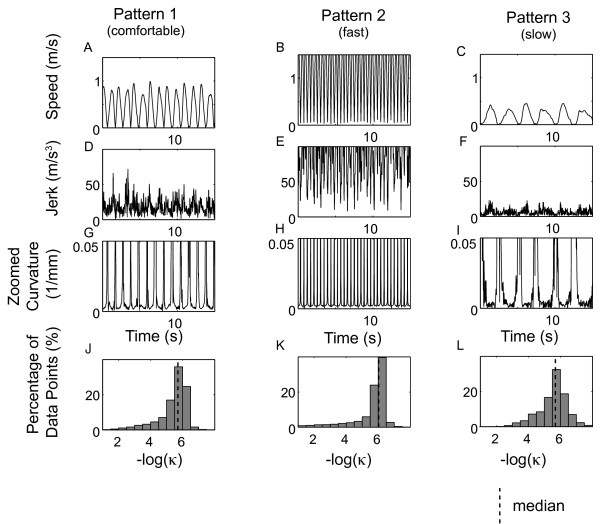
**Speed, jerk, curvature and -log(κ) data for three different movement speeds from the healthy volunteer (H2)**. Panels A, B, and C show the speed; panels D, E, and F show the jerk profile; panels G, H, and I show the zoomed curvature and panels J, K, and L the -log(κ). See Figures 1 and 2 for details.

**Figure 5:**
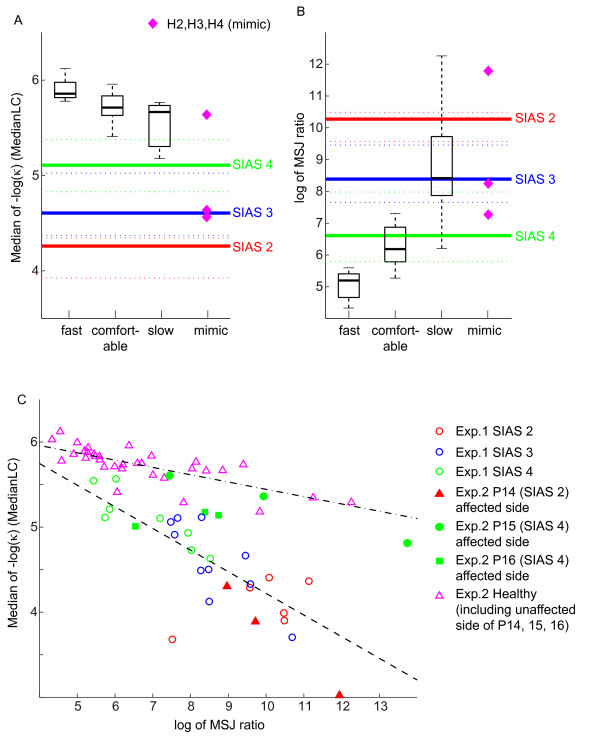
**Comparison between the MedianLC and the log of the MSJ ratio across different movement speeds and SIAS scores**. The boxplots in panels A and B show the median (central marks), the quartiles (edges of the boxes), and the most extreme data points (whiskers) of the MedianLC (Panel A), or the log of MSJ ratio (Panel B) from three different movement speeds (fast, comfortable, and slow) for 11 healthy arm (including three unaffected arm of patients 14, 15, and 16). Magenta diamonds in panels A and B denotes the MedianLC or the log of MSJ ratio from mimicking movements for three healthy participants. Red, blue, and green thick and dotted lines in panels A and B denotes median (thick lines) and quartile (dotted lines) of MedianL from both initial and final measurements in Experiment 1 whose SIAS scores were 2, 3, and 4, respectively. Panel C plots the log of the MSJ ratio against the MedianLC. Magenta triangles denote data from three different movement speeds for 11 healthy arms. Red, blue, and green open circles denote data from initial and final measurements in Experiment 1 where the SIAS scores were 2, 3, and 4. The red filled triangles, green filled circles and green filled squares denote data from three movement speeds for the affected arm of P14 (SIAS score 2), P15 (SIAS score 4), and P16 (SIAS score 4) respectively. The dash dot line shows linear fitting of the data represented by the magenta triangles. The dashed line shows linear fitting of the data represented by the open circles.

Figure 4 shows the speed, jerk, curvature and distribution of the -log(κ) for each movement pattern in a typical healthy participant. Figure 5A shows the boxplots of the MedianLC denoting median and quartile points for each movement pattern. The solid red, blue and green thick line represents the median of MedianLC for SIAS scores 2, 3 and 4 (including both initial and final measurements in Experiment 1), respectively. Although on average there was a 69.5% decrease (SD 13.4%) in peak speed from the fast condition to slow condition (fast condition: mean ± SD of peak speed = 2.72 ± 0.59 m/s; slow condition: 0.82 ± 0.40 m/s), on average the decrease in MedianLC was 5.9% (SD 3.3%). Within these three movement patterns from eleven healthy arms, we observed a correlation between the MedianLC and peak movement speed. However, MedianLC of these three movement patterns from healthy arms was significantly different from that of SIAS score of 4 (Wilcoxon rank sum test, *p *< 0.0001). That is, even when the movement speed was different, we were able to differentiate paretic movements from healthy movements through the MedianLC. Thus, the MedianLC appears to be useful for comparing between normal and irregular movements.

We also examined the sensitivity of the log of MSJ ratio with respect to the movement pattern and speed. Figure 5B shows the boxplots of the log of MSJ ratio denoting median and quartile points for each healthy movement pattern, and median of patient movement for each SIAS score (colored solid lines, see Figure 5A for detail). The log of the MSJ ratio of healthy movements overlapped with that of affected movement, and was not significantly different from that of SIAS score 4. Therefore, it is difficult to differentiate paretic arm movement from healthy movement using the jerk metric if the movement speed is different.

The magenta triangle plots the MedianLC and the log of the MSJ ratio of movement when the healthy participants from the rehabilitation profession mimic the movements of patients affected by stroke. Interestingly, two of the three participants decreased MedianLC to the value comparable to that of SIAS 3 movement, suggesting that they accurately captured the characteristics of movement with a paretic arm. The log of MSJ ratio of these movements was comparable with the value of healthy slow movements.

Figure 5C plots the MedianLC against the log of the MSJ ratio. Although a correlation between the MedianLC and the log of MSJ ratio was observed for the healthy participants (the Spearman ranked correlation coefficients of 0.784, *p *< 0.0001), the slope was significantly different when comparing movements from the healthy participants and the stroke patients (*p *< 0.001). The filled triangles, circles, and squares denote the MedianLC against the log of the MSJ ratio for three movement patterns from affected side of P14, P15, and P16. The MedianLC of movements from patients who scored 4 on the SIAS scale did not differ much among the three movement patterns, as observed in healthy movements, although the log of the MSJ ratio did differ considerably among the movement patterns. However, the MedianLC associated with movement that scored 2 on the SIAS scale, did differ with respect to the different movement patterns, and tended to decrease alongside an increase in speed and a decrease in the MSJ ratio.

### Consistency and reliability of the MedianLC

Using the data from Experiment 1, the consistency and reliability of the MedianLC was assessed using an intraclass correlation coefficient (ICC). To examine consistency within a session, we separated the 15 s data into two 7.5 s components and computed the MedianLC for each component for each participant. We then computed the ICC of the MedianLC between the first half and second half of the measurement. The ICC was 0.949 for the initial measurements and 0.948 for the final measurement, suggesting the MedianLC is highly consistent within a measurement. To confirm the reliability across the sessions, we compared the MedianLC between the initial and final measurements (including in the healthy participant, H1), assuming that the same measurements were repeated under the same conditions for each patient. The ICC was 0.881, which is relatively high. Given that the HANDS therapy (undertaken between the initial and final measurements) led to a change in the SIAS score in three patients, the reliability of the current analysis must be considered to be limited.

## Discussion

In this study, we developed a spatial smoothness measure based on three-dimensional curvature to evaluate movement irregularities in the affected arm of stroke patients. This measure was then compared with clinical assessment scores and with a previously developed measure of smoothness, the MSJ ratio. The measure we developed in this study assessed the median of the natural log of curvature (MedianLC) in the end-point trajectory during three-dimensional reaching. By utilizing this measure, we were able to verify that the SIAS Knee-Mouth test (SIAS K-M), the clinical test used to evaluate clumsiness of the paretic arm in stroke patients, is consistent with the spatial smoothness represented by curvature. The preservation of spatial smoothness during very slow movements in the healthy participant, where temporal smoothness was destroyed, was in contrast with the degradation of spatial smoothness coincident with the loss of temporal smoothness observed in the stroke patients. The measure also correlated with the upper extremity subscale of the Fugl Meyer Assessment that is used to evaluate impairment in stroke patients. Our results show that the MedianLC is a possible tool for evaluating movement quality in the paretic arm of stroke patients.

The MedianLC is not the first method to objectively evaluate the irregularity of movement [17-20,34]. Previous studies have proposed a jerk-based measurement because smoothness in movement is defined as the smallest change in acceleration, which is the definition of jerk [32-35]. Although curvature and jerk differ in the sense that curvature quantifies spatial characteristics, while jerk quantifies the temporal characteristics of trajectory, we found a significant correlation between the MedianLC and the MSJ ratio [27,28]. Since the movements were three-dimensional and required the use of multiple joints (where a greater degree of freedom was allowed), it is reasonable to think that temporal deviation affects spatial deviation and vice versa, and that curvature tends to correlate with jerk. In contrast, the slope was significantly different with respect to the movement observed in the healthy participants and in the stroke patients. For the movements observed in the healthy participants, the MedianLC did not decrease much even when MSJ ratio increased as the participants decreased the speed of their movement. This finding suggests that the quality of paretic movement may be better differentiated by spatial smoothness, represented by curvature, than temporal smoothness, represented by jerk, if the movement speed is uncontrollable. Although in single case, we observed a coincident reduction of spatial and temporal smoothness when movement speed increased in a patient with a SIAS score of 2. This finding was not observed in the two patients with a SIAS score of 4. Further research is necessary to resolve the relationship between severity of impairment, movement speed and movement irregularity.

Spatial irregularity has previously been evaluated by measuring the ratio of actual hand path and direct path length (represented as an index of curvature, IOC) [21-23]. The IOC measures the degree of deviation in hand path in one whole movement segment. In contrast, the metric described in this study quantifies instantaneous curvature at each time point. In the current data, we did not find significant correlation between IOC measure and MedianLC. This may partly be because the IOC cannot separate between hand paths characterized by less meandering than those with more meandering if the path length of the two is the same.

An advantage of the MedianLC over a jerk-type measure or IOC is that movement segmentation is not required. As a jerk measure has to be normalized with respect to both duration and amplitude of each segment of the movement, it is very important to identify each segment that includes a single stroke. The IOC measure also requires the direct path length of each segment. However, for patients, it is often difficult to clearly identify the timing of movement initiation and termination because of the irregularity of the movement [29]. Therefore, MedianLC is advantageous for the analysis of paretic arm movements.

The reliability of the MedianLC was confirmed by calculating the ICC between the two measurements, at approximately 2 weeks apart, although the reliability of the result is limited by the intervention between the two measurements. Consistency within a measurement was also assessed by the high ICC between the first and the second 7.5 s block of data in each measurement. However, in some patients we observed a difference in the MedianLC between the first half and the second half (mean ± SD of the difference was 0.14 ± 0.13). Since the 15 s data was halved without accounting for movement segments, an incomplete segment may have caused measurement noise. To acquire a more consistent MedianLC, a longer analysis time window would be preferable. On the other hand, there is the possibility that the patients' performance itself might have actually changed during a measurement. For instance some patients showed an increased MedianLC in the second half, suggesting the possibility of practice effect, while some others had a reduced MedianLC, possibly because the patients were tired or their movements became more spastic. The MedianLC is most reliable when the movement is consistent throughout a measurement and there is a long enough duration for analysis (15 s or more). However, for patients a shorter measurement period is preferable, and the shortest minimum duration that gives the most reliable values must be taken into account when transferring this type of metric to the clinic.

The relationship between the MedianLC and the clinical observation of clumsiness was assessed by determining the correlation between the MedianLC and SIAS K-M. These two variables highly related. The initial MedianLC was correlated with the initial SIAS K-M and the final MedianLC was correlated with the final SIAS K-M. Five out of three patients with a SIAS score of 2 at admission improved to a SIAS score of 3 at discharge [9]. However, the MedianLC value did not increase significantly in these patients and their MedianLC at discharge was 4.13, 4.61, and 3.71, respectively, which was smaller than the average MedianLC of the SIAS 3 group at initial measurement (MedianLC value of 4.76). This may be because the transition from SIAS 2 to SIAS 3 is not based on smoothness, but on the ability to reach the hand high enough, and the improvement in spatial smoothness was not in parallel with the promotion to the SIAS score of 3 from 2. The correlation between MedianLC and the clinical assessment of FMA-UE, on the other hand, demonstrates that the movements with less spatial irregularity result in better upper extremity function. Therefore, MedianLC represents a useful indicator of the functional recovery in the upper extremity.

Even within the group with the same SIAS K-M score, some variability of the MedianLC was observed. Because the ICC across measurements was relatively high, the MedianLC may be a finer scale of movement irregularities than the expert rating. Also, given that MedianLC does not require an expert's observation, if the measurement system were to be made portable and easy to use, it could be used as a self-training system feedback mechanism available to patients for daily rehabilitation. Patients could learn smoother movements by trying to increase the score in the movement training.

Computationally, smoothness has been discussed as a candidate objective function that should be optimized at the trajectory planning level. In contrast, the mechanism that increases curvature in stroke patients would not be limited to the degradation in trajectory planning. Degradation in the internal model [38,39], distortion in the feedback including sensory deficits, a reduction in motor command [40], or an increase in motor command noise, can lead to an increase in curvature. Any inappropriate increase in mechanical impairments due to spasticity or an increase in tone may also cause movement irregularities. In the present study, we did not find a significant relationship between the MedianLC and the modified Ashworth scale (Table 2). It is possible that the variation in the MedianLC within each SIAS score group was due to the level of spasticity; however, further investigation is required to fully investigate these issues.

## Conclusions

In this study we developed a measure of spatial smoothness based on three-dimensional curvature that was effective in evaluating movement irregularities in the affected arm of stroke patients. The measure presented in this report assesses the median of the natural log and was comparable to an examiner's observation, as well as to a clinical assessment of functional recovery. The results of this study suggest that the quality of paretic movement is characterized through spatial smoothness represented by curvature. The smaller computational cost involved in acquiring this measurement suggests that this method may be use a useful tool in clinical settings.

## Competing interests

The authors declare that they have no competing interests.

## Authors' contributions

RO performed analysis of data and drafting of the manuscript. KO performed the design of the experiments and executed experiments. TF made substantial contribution to acquisition of the data and recruitment of the patients. YO, MK, and ML were involved in the interpretation of the results and critical revision of the manuscript. All authors read and approved the final manuscript.

## Appendix

### The Stroke Impairment Assessment Set (SIAS)

The SIAS is a comprehensive instrument used to assess stroke impairment, which provides information on motor function, tone, sensory function, range of motion, pain, trunk function, visuo-spatial function, speech and sound side function. The SIAS test can also be used to separately assess the proximal and distal upper extremity motor function.

#### Proximal Upper Extremity motor function test (Knee-Mouth test)

The application of the SIAS test to measure upper extremity function can be performed as follows. In the sitting position, the patient touches the contralateral knee with the affected hand and then lifts the hand to the mouth. When the hand reaches the mouth, the affected-side shoulder is abducted to 90 degrees. Then, the hand is returned to the knee. The test is performed three times. If contracture of the shoulder or elbow is present, the test is judged on the basis of movement within the range of motion. The score is based on the following criteria:

0 = There is no contraction of biceps brachii.

1 = Minimal voluntary movement is noted, but the patient cannot raise the hand to the level of the nipple.

2 = Synergic movement is noted in the shoulder and elbow joints, but the patient is not able to touch the mouth with the affected-side hand.

3 = The patient carries out the task with severe or moderate clumsiness.

4 = The patient carries out the task with mild clumsiness.

5 = The patient carries out the task as smoothly as on the unaffected side.

### Motion capture system

OPTOTRAK Certus is a motion capture system that can acquire high-frequency three-dimensional position data with an accuracy of up to 0.1 mm and resolution of 0.01 mm. From the LED marker attached to the glass, infrared light was emitted, which was detected by three cameras.
